# Understanding the impact of visual arts interventions for people living with dementia: a realist review protocol

**DOI:** 10.1186/2046-4053-3-91

**Published:** 2014-08-15

**Authors:** Gill Windle, Samantha Gregory, Andrew Newman, Anna Goulding, Dave O'Brien, Clive Parkinson

**Affiliations:** 1 Dementia Services Development Centre, Bangor University, Ardudwy, Holyhead Road, Bangor, Gwynedd LL57 2DG, Wales; 2 International Centre for Cultural and Heritage Studies, Newcastle University, 18 Windsor Terrace, Newcastle on Tyne NE1 7RU, England; 3 Centre for Cultural Policy and Management, City University London, London EC1V 0HB, England; 4 Manchester Metropolitan University, Righton Building, Cavendish Street, Manchester M15 6BG, England

**Keywords:** Realist synthesis, Realist review, Art interventions, Art programmes, Creative activities, Dementia, Visual art, Imagination

## Abstract

**Background:**

Arts-based activities are being increasingly suggested as a valuable activity for people living with dementia in terms of countering the negative aspects of their condition. The potential for such programmes to improve a broad range of psychosocial outcomes is suggested in some studies. However, there is largely an absence of rigorous methodology to demonstrate the benefits, and research results are mixed. Practice variability in terms of the content, contexts and implementation of such interventions raises challenges in terms of identifying an optimal arts programme model that could be adopted by other service providers. Understanding how interventions may have the best chance at broad implementation success and uptake is limited.

**Methods/Design:**

A realist review will be undertaken. This aims to understand how visual arts interventions influence outcomes in people living with dementia. The review will explore how the context, that is the circumstances which enable or constrain, affect outcomes through the activation of mechanisms. An early scoping search and a stakeholder survey formulated the preliminary programme theory. A systematic literature search across a broad range of disciplines (arts, humanities, social sciences, health) will be undertaken to identify journal articles and grey literature. Data will be extracted in relation to the programme theory, contextual factors, mechanisms and outcomes and their configurations, background information about the study design and participant characteristics, detail about the quantity (‘dose’) of an intervention, theoretical perspectives proposed by the authors of the paper and further theorising by the reviewer. Thematic connections/patterns will be sought across the extracted data, identifying patterns amongst contextual factors, the mechanisms they trigger and the associated outcomes.

**Discussion:**

Along with stakeholder engagement and validation, this review will help inform the development of an optimal, replicable arts intervention for people with dementia as part of our broader research programme, titled ‘Dementia and Imagination’ (funded by the Arts and Humanities Research Council). Forthcoming work under this programme of research will test this theoretically informed intervention in three different geographical areas of the UK. The production of freely available practice guidance is a key aspect of dissemination.

**Trial registration:**

PROSPERO registration number CRD42014008702.

## Background

### Rationale

There is no cure for dementia; therefore, attempts to maintain quality of life and well-being are crucial. Dementia is now firmly on the international public and policy agenda, bringing opportunities for change on a wider scale for those living with the condition [1] and the communities in which they live. Changing awareness and understanding about dementia and enabling people to ‘live well’ is central to the National Dementia Strategy [2]. Creating dementia-supportive or dementia-friendly communities is a major policy objective and a societal imperative noted in both the National Dementia Vision for Wales [3] and the UK Prime Minister's Challenge on Dementia.

The cultural representation of dementia has a stigmatising effect on everyday interactions both for the person with dementia [4] and their carer or partner. A survey of over 2,000 members of the general public reports that 39% of respondents perceived people with dementia in the UK to have a fairly bad quality of life, with 19% reporting a very bad quality of life [1]. Another concludes that the general public often has a narrow and negative way of thinking about people with dementia [5]. In the face of this adversity, the dementia community often becomes disconnected, marginalised and excluded from society. Many people with dementia have minimal or no access to activities usually regarded as normal, and those that do exist are often much below the person's level of functioning [6]. Yet people with dementia wish to participate in activities, feel valued and have a sense of purpose [7].

Arts-based activities are being increasingly suggested as a valuable activity for people living with dementia in terms of countering the negative aspects of their condition. They represent a range of activities, including dance, music, creative writing, visual art and singing. Attempts at demonstrating the benefits for people with dementia of creative engagement in art and cultural programmes have been undertaken in a limited number of peer-reviewed research studies and have been summarised in four reviews [8-11]. The potential for such programmes to improve a broad range of outcomes such as well-being, quality of life, cognitive function and creative thinking; increases in communication (including non-verbal), facilitating reminiscence and meaningful conversation; regaining a sense of self; increasing self esteem; and improving the quality of life of carers is suggested in some studies.

Despite the apparent potential of art, the reviews identify a major research gap; on the whole, although the scientific base is improving, there is largely an absence of rigorous methodology to demonstrate the benefits, and research results are mixed. This variability has been attributed to factors such as small sample sizes and inadequate research design, including methods of data collection on the one hand and differences in intervention content and delivery on the other. In practice, complex interventions such as these are often implemented in a diverse manner by different stakeholders (who will have varying levels of skills), to diverse populations and in different settings, all of which can affect the outcome of the programme [12]. This variability raises challenges in terms of identifying an optimal arts programme model that could be adopted by other service providers for implementation in further practice. What ‘works’ in one setting may not have the same benefits in others.

A further challenge in understanding how an arts intervention works, for whom and in which contexts is that to date, there has been little research or theorising in this area that attempts to explain the underlying theoretical mechanisms/processes through which the benefits of participation in arts activities develop and are sustained [9,13]. This is important for understanding how interventions may have the best chance at broad implementation success and uptake.

This review will address some of the current limitations through the development and subsequent testing of a ‘programme theory’ for an arts intervention, using realist review methodology. This programme theory aims to identify how, why, for whom and in what contexts art interventions for people with dementia may be more likely to be effective or in which circumstances they may not lead to positive outcomes.

This work is an important first step to help inform the development of an optimal, replicable arts intervention for people with dementia as part of our broader research programme, titled ‘Dementia and Imagination’. This is a novel and challenging application of realist methodology. Forthcoming work under this programme of research (not reported here) will test this intervention in three different geographical regions in England and Wales.

The Dementia and Imagination research programme (and this review) focusses on visual art. There is currently no evidence to suggest that one art form is more appropriate or beneficial than another. The Mental Health Foundation review [10] notes that a current limitation of the evidence base is that very few studies have focussed on one art form, and consequently, it is not possible to demonstrate the strength of the evidence through the cumulative effects of a number of related studies (p. 35). To address this, the review contributes to the development of a theoretically informed visual arts programme.

Given the increasing numbers of people living with dementia, the potential for non-pharmacological approaches such as arts-based activities that can help people live well with the condition have considerable public health implications.

### Objectives and focus of the review

The realist review approach is an iterative process, aimed at uncovering the theories that inform decisions and actions [14]. A systematic review can inform as to whether an intervention may (or may not) be effective, but does not look at how and why effectiveness may occur, and the extent to which the context may, in the language of realist methodology, trigger different processes or mechanisms to influence outcomes. This is addressed through a realist review approach to evidence synthesis. The aim of this review is to understand how visual arts interventions influence outcomes in people living with dementia. To realise this aim, the review will explore how contexts, that is the circumstances which enable or constrain, affect outcomes through the activation of mechanisms. This will be undertaken as follows:

1. Initial programme theory development

2. Searching, retrieval, data extraction and synthesis, testing and refining the programme theory

3. Production of evidence to inform the development of a visual arts intervention for people with dementia

### Review questions

● How and why are there changes in the outcomes of interest—what are the mechanisms through which the identified visual art programmes influence outcomes?

● What are the contexts/conditions which determine whether the different mechanisms influence outcomes?

● In what circumstances (which combinations of mechanisms and contexts) are visual arts interventions most likely to be effective?

These questions were developed through a number of iterations (discussed in the ‘Methods/design’ section) in order to focus the purpose of the review. The team recognise these questions may require further modification as the review progresses, should stakeholder input or theoretical lines of inquiry suggest new pathways. Such approaches to iterative refinement are recommended for focussing reviews [15].

## Methods/Design

The starting point for our programme theory was initial concept mining and theory formulation about how and why the interventions might be effective. This was an iterative process and consisted of initial exploration of some of the literature (journal articles and grey literature) by members of the team who were familiar with the topic and in contact with broader stakeholder networks (the work programme has over 50 organisations/individuals who have asked to receive information or be involved in the research programme) and discussion meetings within the team (the multidisciplinary team reflect the arts and humanities, health, psychology and gerontology). This was initiated through posing an initial question—what is it about visual arts interventions that make them work? How are they effective? The team also discussed and defined key terms to guide the review, to ensure a common understanding (see Additional file 1).

**Figure 1:**
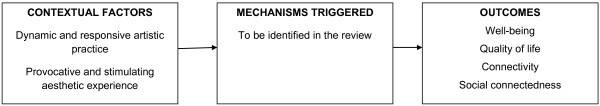
Preliminary programme theory.

A regular aspect of a realist review is the involvement of stakeholders, particularly when broader expertise on the content of interventions is important. In this research, stakeholders who had taken part in or been involved in delivering visual art interventions were invited to contribute to programme theory development through a survey (online and paper) which was circulated widely through the practice networks of team members and the Dementia and Imagination stakeholders mail list. All recipients were asked to forward onto others in their own networks. The aim of the survey was to build an understanding of the topic area through utilising the stakeholder perspectives to identify how and why arts programmes are effective. The survey was simplified to ask people to give up to ten ‘tips’ from their own experiences for what made the arts intervention/activities good and also opinion on when they did not work well. Thirty-seven people responded (6 people with dementia, 5 carers, 26 service providers/artists; results will be reported elsewhere).After a number of iterations and discussions, we developed an initial but basic programme theory regarding how and why visual arts interventions might work. This suggests that visual arts interventions should be built on dynamic and responsive artistic practice (e.g. good skills and understanding) and they should create a provocative and stimulating aesthetic experience (e.g. be challenging and engaging, in an inspiring environment) which triggers the mechanisms that lead to well-being, quality of life, connectivity and social connectedness (see Figure 1).

### Searching process

In the first instance, a broad literature search will be undertaken within the following databases: ASSIA, Medline, SAGE, CINAHL, PsychInfo, PsycARTICLES, Sociological Abstracts, Web of Knowledge, JSTOR and the Arts and Humanities databases within ProQuest to ensure a broad range of sources across disciplines are addressed. Search terms have been developed iteratively (through piloting and refinement) between two researchers (GW and SG) using combinations of keywords (see Additional file 2). The searches are deliberately designed to ensure a focus on the review questions and the initial thinking around the programme theory; however, no restrictions are imposed on the type of study designs selected. The searches will be undertaken by a member of the team (SG) and will focus on identifying primary research, published in English, from 2000 onwards. The date is a pragmatic choice, based upon the time limit and resources available for completing the review. The selection and appraisal procedure is described in the following section.

All team members will review the final list of titles to ensure potentially relevant papers are not missed by the search strategy. Following the initial searches, ongoing updates will be periodically undertaken as will new searches to identify further data to (a) test in relation to the existing programme theory, (b) develop and refine the programme theory and (c) identify substantive theory to clarify the programme theory.

Grey literature will be located through a number of methods. An initial Google search to identify relevant community arts and dementia programmes has been undertaken, and details including the web address have been recorded. These will be searched for any relevant evaluations/reports, and organisations may also be contacted for further information. Source documents will be used to locate schemes; contacts through existing networks will seek further sources of information.

### Selection and appraisal of documents

This will be undertaken in three stages: (1) a preliminary screen, (2) full text retrieval and (3) appraise and include. At the screening stage, the researcher (SG) is guided by the inclusion/exclusion criteria which are broad enough to identify a wide range of material (an inclusive approach). Papers will be included for the next stage if they are primary research conducted with older adults and/or people with dementia; describe interventions that relate to art, creativity, community, museums, galleries, hobbies and learning; were published in English, from 2000 onwards; and were about participatory art and community art. Papers will be excluded in the first stage if the research was of no relevance to Alzheimer's disease/dementia; they were pharmacological intervention studies; they adopted only neurological outcome measures such as MRI or EEG (not relevant to the outcomes of interest in Dementia and Imagination); and they focussed on art therapy. If there is any ambiguity, the reviewer will include the title for further appraisal. A proportion of the references (30%) will be scanned in duplicate by a second reviewer (GW). To minimise discrepancies, both reviewers worked closely on the piloting stage. However, any discrepancies will be discussed between the two reviewers and referred to a third member of the team if necessary. Excluded documents will be saved. A document will be created with the reference, abstract and reason for exclusion. This could be consulted to enable further identification of relevant data, should any modifications occur as the programme theory develops.

All search results will be saved in RefWorks, an online reference manager, which is backed up on a secure server at the university.

In contrast to a systematic review, which judges the quality of the research according to the rigour of the design and methods, a realist review aims to examine whether it is fit for purpose according to relevance and rigour. With this in mind, two questions are used to guide the appraisal of papers.

● Relevance—does it contribute to theory building and testing?

● Rigour—are the methods used to generate the relevant data credible and trustworthy? Source [15].

Studies will not be excluded according to their design. As described by Pawson, ‘an otherwise mediocre study can indeed produce pearls of explanatory wisdom’ [16], p. 9. However, given the lack of larger research studies and randomised controlled trials in this research area, in line with the RAMESES quality standards for realist synthesis [15], the methodological limitations of any studies included in the review (rigour) will be identified and considered during analysis and synthesis.

Each paper will be reviewed by two members of the team. Any disagreements will be resolved through discussion within the review team, and if necessary, the research external advisor (JRM) will be consulted for advice. All excluded papers will be retained for future appraisal if necessary.

### Data extraction

In order to identify what might constitute the active ingredients of a successful visual arts intervention in what context, a bespoke data extraction document will be used. This is designed to answer the research questions and extract specific information in relation to the programme theory, mechanisms and outcomes, background information about the study design and participant characteristics, detail about the quantity (‘dose’) of an intervention, theoretical perspectives proposed by the authors of the paper, further theorising by the reviewer, contextual factors and context-mechanism-outcome (CMO) configurations. Direct quotations will be included. This will be in Word format and will initially be piloted to ensure its relevance to the programme theory and modified if necessary.

The team developed a list of questions to guide their thinking whilst undertaking data extraction and help identify mechanisms (e.g. Do the techniques involve new learning?). If information is missing, the team will note this as ‘not reported’. Each paper will be examined by two reviewers independently. Data extraction will be undertaken by one member of the team, with a second person cross-checking this initial work by undertaking a second appraisal of the paper, identifying any inconsistencies, errors or omissions and amending where necessary in a different colour text for identification. Any amendments will then be discussed between the two reviewers, and agreement sought more broadly within the review team if necessary.

### Analysis and synthesis process

Data analysis and synthesis will be undertaken by a lead reviewer, and synthesis results will be regularly shared and discussed within the review team and with stakeholders to ensure they are valid and meaningful.

The research questions will be used to guide the analysis and synthesis. The analysis aims to iteratively test and refine the programme theory using empirical findings in the included papers. Realist principles will be embedded in the analysis. To facilitate this phase, a central database (using Excel) will be created. This will take the data/sections of text from the extraction phase and place them into one of three categories (context, mechanism, outcome). Propositional statements will be structured as context-mechanism-outcome configurations for each paper. Following the rationale of Jagosh et al. [17], the reviewer will record any reflections or interpretations of the evidence.

Following this, thematic connections/patterns across the identified outcomes will be sought. For example, comparisons between contextual factors hypothesised to be important in relation to the programme theory will be made. Patterns of similar mechanisms will be compared across different contexts to see if similar outcomes are generated. Cases where the contexts have been constraining, rather than enabling, will be identified. In realist terms, these are described as ‘demi-regularities’ and describe semi-predictable patterns of the functional aspects of the programme [17]. The programme theory will be amended if necessary if new CMO configurations arise.

Given the nature of the topic under question (older people, dementia, art), existing explanatory theories (described as formal theory or substantive theory in realist review) can be drawn from a range of different academic areas. These can further help understand the CMO patterns and contribute to the synthesis [15]. Three relevant examples are presented below.

The substantive theory of relationship-centred care led to the Senses Framework [18], which suggests that all those involved in caring (e.g. the person living with dementia, family carers and other staff involved in providing services) should experience relationships that promote a sense of security, belonging, continuity, purpose, achievement and significance in order for services to be appropriate and life enhancing;

The constructionist museum model [19] is based on the co-construction of knowledge between the museum or gallery curator and visitor (or in this case, between the intervention facilitator and the participant). This helps to explain how learning takes place within a museum or gallery environment.

Kroger's identity revision and maintenance processes (how individuals could maintain or revise a sense of who they are within their immediate and broader social networks and contexts) [20] might be used to determine how encounters with art help people with dementia re-establish a sense of self that might have been lost or to create a new sense of self (as far as the illness might allow). Using this body of work, an optimal sense of identity is experienced as a subjective sense of sameness and continuity across time and space [21] which could be fractured by the effects of the condition (biographies would be disrupted and life review or reminiscence made difficult or even impossible). Encounters with art (viewing and making) might be seen as playing an important part in the above because it facilitates communication and collectivises feelings that are not easily addressed otherwise.

The findings will be synthesised to be of practical use. One application of the findings will be to inform the development of a forthcoming visual arts intervention which will be tested in our ongoing work programme. This practical application will enable further refinement of the programme theory and ultimately establish an evidence-based account of how visual arts programmes for people with dementia are effective.

### Reporting and dissemination of findings

In reporting the research, we will follow the methodological framework developed from Pawson [12] by Rycroft-Malone et al. [22] and the RAMESES quality standards [15] and adopt the RAMESES publication standards [23]. An academic article reporting the findings of this review will be written for publication in an international journal.

The findings also have a practical use, informing the Dementia and Imagination research intervention, which will be tested in the forthcoming programme of research and will form the basis of a number of research papers. In terms of broader communication and knowledge sharing, the research team has developed a communication strategy which identifies a large range of target stakeholders and their requirements. The research team also has a mailing list of associated practitioners/stakeholders who have expressed interest in the study as it has been developing from the research idea to a fully funded study. The findings will also be disseminated through formal discussions/conferences with stakeholders and academics and through the project website.

### Trial status

The review has been registered with PROSPERO, the international register of systematic reviews: CRD42014008702.

## Discussion

This review aims to unpick some of the complexities around how and why visual arts interventions for people with dementia, potentially implemented in a diverse manner by different stakeholders in diverse settings, might succeed in achieving positive outcomes. Through this realist methodology, a programme theory which identifies the relationships between contexts, mechanisms and outcomes will be developed. Along with other methods (stakeholder discussion and survey), this will inform the development of an optimal, replicable arts intervention for people with dementia, and future work under this programme of research (not reported here) will test this intervention in three different geographical locations and produce practice guidance.

In terms of the limitations of this work, a major challenge to the theory building will likely be in relation to the rigour of the relevant research. To address this, the team will consider the findings in relation to the way each of the included studies are conducted and reported, reflecting the credibility and trustworthiness, recognising any limitations in the final synthesis and subsequent recommendations.

It is acknowledged that the focus of this review will not produce a definitive answer, and other theoretical approaches to understanding how visual arts interventions produce their effects on health and well-being could be posited. However, the initial theorising involved a multidisciplinary team (arts, humanities, health, psychology, gerontology) which helps ensure a broad range of theoretical perspectives are considered. A range of different stakeholders have been involved in the work, and the next steps will involve engagement with these and the Dementia and Imagination artists in terms of receiving comments on the validity of early findings and in the development of the research intervention.

## Abbreviations

RAMESES: Realist And Meta-narrative Evidence Syntheses: Evolving Standards; CMO: context, mechanism, outcome.

## Competing interests

The authors declare there are no competing interests.

## Authors’ contributions

GW led the development and writing of the protocol. SG contributed to early drafts of the protocol and identified literature for the scoping work. SG initiated early theorising about the programme theory. GW, SG, AG, CP, AN and DOB developed the programme theory. All authors read and approved the final manuscript.

## Authors' information

GW (BSc; MSc; PhD) is a senior research fellow in gerontology and lead investigator of the Dementia and Imagination research programme. SG (BSc; MSc) was a research assistant with GW and is now a PhD student. AN is a senior lecturer in Museum Studies at Newcastle University and is a Co-I on Dementia and Imagination. He is interested in the consumption of art by older people. AG is a research associate at the International Centre for Cultural and Heritage Studies, Newcastle University, who has looked at the impact of engaging with art on older people through a range of projects. DO'B (BA; MA; PhD) is a lecturer in Cultural and Creative Industries at City University London. He specialises in cultural value and urban cultural policy issues. CP is the Director of Arts for Health at Manchester Metropolitan University.

## Supplementary Material

Additional file 1 **Definitions (revised.docx).** This file contains key terms used by the review team. Click here for file

Additional file 2 **Search terms (revised.docx).** This file contains the search terms used to identify the literature. Click here for file
