# Supplementary material for: Understanding the impact of visual arts interventions for people living with dementia: a realist review protocol
Source: Syst Rev. 2014 Aug 15;3:91. doi: 10.1186/2046-4053-3-91 (PMC4141269; doi:10.1186/2046-4053-3-91)
Supplement: Additional file 1 — Definitions (revised.docx). This file contains key terms used by the review team. [file 2046-4053-3-91-S1.docx]

**Additional file 1: Definitions**

| **Methodological definitions** |  |
| --- | --- |
| Realist synthesis/realist review | A realist synthesis aims ‘to articulate underlying programme theories and then to interrogate existing evidence to find out whether and where these theories are pertinent and productive. Primary research is examined for its contribution to the developing theory…(Pawson, p.74, 2006).  The rationale behind a realist synthesis is that an intervention conducted by a certain group of people, with a certain group participants in a specific setting may not always work the same in a new setting, with new people. This is because people are active participants in the world. Therefore the realist review looks at an intervention as a series of theories implemented together, it looks at who is implementing the theories and in what circumstances (context) what effect this has (outcomes), and how the context impact on the outcomes through identifying mechanisms of change. This approach is important when dealing with complex programmes where, due to their complexity, the programmes are implemented in a diverse manner by different stakeholders, to diverse populations and in diverse settings, all of which can affect the outcome of the programme.  Pawson, R. (2006). Evidenced-based policy: A realist perspective. London: Sage. |
| Context | The circumstances that form the setting for an event, statement, or idea, and in terms of which it can be fully understood (http://oxforddictionaries.com/definition/english/context)  According to Pawson et al (2004) the main contextual factor that shapes an intervention are the ‘existing social systems that are thought to underpin and account for present problems’(p4) A key requirement of realist enquiry is thus to take heed of the different layers of social reality that make up and surround interventions’ (p7 Pawson et al 2004)  Pawson et al (2004) highlights a further 4 contextual factors that should be considered:  (a) The individual capacities of the key actors and stakeholders.  (b) The interpersonal relationships required to support the intervention.  (c) The institutional setting.  (d) The wider infra-structural and welfare system.  Pawson, R et al. (2004). Realist Synthesis, an introduction. ESRC Research Methods Programme. Available online at: <http://www.ccsr.ac.uk/methods/publications/documents/RMPmethods2.pdf> |
| Mechanisms | A natural or established process by which something takes place or is brought about (<http://oxforddictionaries.com/definition/english/mechanism?q=mechanism>)  ‘Mechanisms generate outcomes’ (Wong et al., 2013, p.14).  ‘Mechanisms are the agents of change. They describe how resources embedded within a programme influence to reasoning and ultimately the behaviour of programme subjects (Pawson, 2013, p.13)  Pawson, R. (2013). The Science of Evaluation: A Realist Manifesto. London: Sage.  Wong G, Westhorp G, Pawson R, Greenhalgh T. **Realist synthesis. RAMESES training materials***.*  [.http://www.ramesesproject.org/media/Realist_reviews_training_materials.pdf]  . |
| Outcomes | The outcomes come from the interaction of the mechanisms and the context (Pawson et al 2004). Outcomes can be positive or negative. They are the results of the intervention. |
|  |  |
| **Outcomes** |  |
| Connectivity | Connectivity is defined as the link between individuals, groups or objects through some pattern, process or structure (Means, Burholt & Hennessey, 2012).  Means, R., Burholt, V. Hennessy, C. (2012). Exploring Connectivity and Rural Ageing: Issues and Challenges from ‘the Grey and Pleasant Land?’ (GaPL) Project. Presented at the BSG annual conference, Keele, 11^th^-13^th^ July. |
| Social connectedness | The experience of belonging and relatedness between people |
| Well-being | There is no single definition of well-being. From the literature it can be summarised that:   - Well-being is synonymous with positive mental health - Well-being encompasses hedonic (e.g. happiness) and eudaimonic (e.g. purpose in life, achieving potential, personal growth) aspects of mental health   Participants in phase one, Voices on wellbeing, of the Shaping our Age project (2011) describe the feelings of well-being as: happiness, contentment, satisfaction, peace of mind, comfort, enjoyment and euphoria. Well-being is also associated with feelings of self-worth and achievement.’  The Greater Cincinnati Chapter Well-Being Observation Tool is used to assess well-being in people with dementia (Kinney & Rentz 2005). This assesses the seven domains of well-being among individuals with dementia. These seven domains are:  Interest, sustained attention, pleasure, negative affect, sadness, self-esteem, and normalcy.  Kinney, J. M., & Rentz, C. A. (2005). Observed well-being among individuals with dementia: Memories in the making©, an art program, versus other structured activity. *American Journal of Alzheimer's Disease andOther Dementias*, 20(4), 220-227. |
| Quality of life for people with dementia | According to a report on research by the Alzheimer’s Society (2010, My name is not dementia) the key quality of life indicators and findings for people with dementia, in order of importance are:  1. Relationships or someone to talk to  2. Environment  3. Physical health  4. Sense of humour  5. Independence  6. Ability to communicate  7. Sense of personal identity  8. Ability or opportunity to engage in activities  9. Ability to practise faith or religion  10. Experience of stigma  Importantly, they found that ‘*People with dementia have a clear need for regular, everyday, one to one social interaction and this will have significant benefit to their quality of life and social inclusion.’* (Alzheimer’s Society 2010)  Alzheimer’s Society. (2010). My name is not dementia: people with dementia discuss quality of life indicators. Available online at: <http://www.alzheimers.org.uk/site/scripts/download_info.php?fileID=876> |
| **Art descriptions** |  |
| **Art therapy** | Art therapy is a form of therapy that uses art as communication, rather than for the purpose of actual creativity and enjoyment (Darton, 2012).  [This is not what we are looking at as part of this review, however, the term is often used when describing the types of interventions that we are addressing. Therefore the term art therapy will not be screened out during the literature search process (unless it can clearly be derived from the abstract that the intervention is indeed Art therapy), and instead will be addressed at the data extraction stage].  Darton, K. (2012) making Sense of Arts Therapies. Mind (National Association for Mental Health) |
| **Visual art** | The Collins English dictionary describes visual art as: the arts of painting, sculpting, photography, etc, as opposed to music, drama, and literature (Collins, 2013). Thus, visual art refers to art work that has a form, it refers to something that is created that we can look at.  Collins, (2013) http://www.collinsdictionary.com/dictionary/english/visual-arts |
| **Participatory art** | For the art to be participatory there must be participation, the people involved must work with the artist, rather than be passive observers.  ‘*Participatory arts are by their very nature collaborative. The professional skills of the artist combine with the creative energy of the participants to produce an event or an experience that is in many ways more than the sum of its parts.*’ (Dix & Gregory 2010 p. 13)  Dix, A. and Gregory, T. (2010) Adult participatory arts. Thinking it through. A review commissioned from 509 Arts for Arts council England |
| **Creative activity** | Creativity is defined by the oxford dictionary as: the use of imagination or original ideas to create something; inventiveness (<http://oxforddictionaries.com/definition/english/creativity>)  However it is not as simple as that and when you start to look you can find many alternative views from many fields on what constitutes creativity, with internet pages dedicated to collating these ideas (<http://celestra.ca/top-10-creativity-definitions/> ; <http://www4.uwsp.edu/education/lwilson/creativ/define.htm> ; <http://www.csun.edu/~vcpsy00h/creativity/define.htm>)  In an article on developing creativity in children, Caroline Sharp has condensed the various ideas on defining creativity into a 5 bullet point list:  • Imagination  • Originality (the ability to come up with ideas and products that are new and unusual)  • Productivity (the ability to generate a variety of different ideas through divergent thinking)  • Problem solving (application of knowledge and imagination to a given situation)  • The ability to produce an outcome of value and worth.  (Sharp, C. (2004) Developing young children’s creativity: what can we learn from research? *Topic* 32 5-12)  Thus a creative activity should be activity where the participants use their imaginations to create original work that they consider to be of worth due to them having put something of themselves into it. |
| **Socially engaged arts practice**  (this is the central premise of what we want to do) | Summarised from Froggett et al. 2011:  Facilitated through the process of developing artwork; those that deliver focus on process and seek to embed themselves within the communities in which they work; responsive to the needs of the communities; stimulates forms of connectivity, as socially engaged arts practice builds links between temporary or permanent communities of place, interest or practice.  Froggett, L., Little, L., Roy, A. & Whitaker, L. (2011). New model visual arts organisations and social engagement.Available online at: <http://www.uclan.ac.uk/schools/school_of_social_work/research/pru/files/wzw_nmi_report.pdf> |
