# Supplementary material for: Understanding the impact of visual arts interventions for people living with dementia: a realist review protocol
Source: Syst Rev. 2014 Aug 15;3:91. doi: 10.1186/2046-4053-3-91 (PMC4141269; doi:10.1186/2046-4053-3-91)
Supplement: Additional file 2 — Search terms (revised.docx). This file contains the search terms used to identify the literature. [file 2046-4053-3-91-S2.docx]

**Additional file 2: Keywords for searches**

1. **Target population**

Dementia; Dementia care; Older adults; Elder; age*; Alzheimer’s Disease

1. **Intervention**

Creative; Creativity; Art; Community; Participatory; Activity; hobbies; galleries; meaningful activity; visual art;

1. **Outcomes**

Quality of life; well-being; self-esteem; memory; health; social; relationships; social connections; connectedness.
